# Supplementary figures and images for: A MeA Tac1 neural circuit mediates anxiety-like behaviors in mice
Source: EMBO Rep. 2025 Jul 28;26(17):4340–63. doi: 10.1038/s44319-025-00528-z (PMC12420787; doi:10.1038/s44319-025-00528-z)

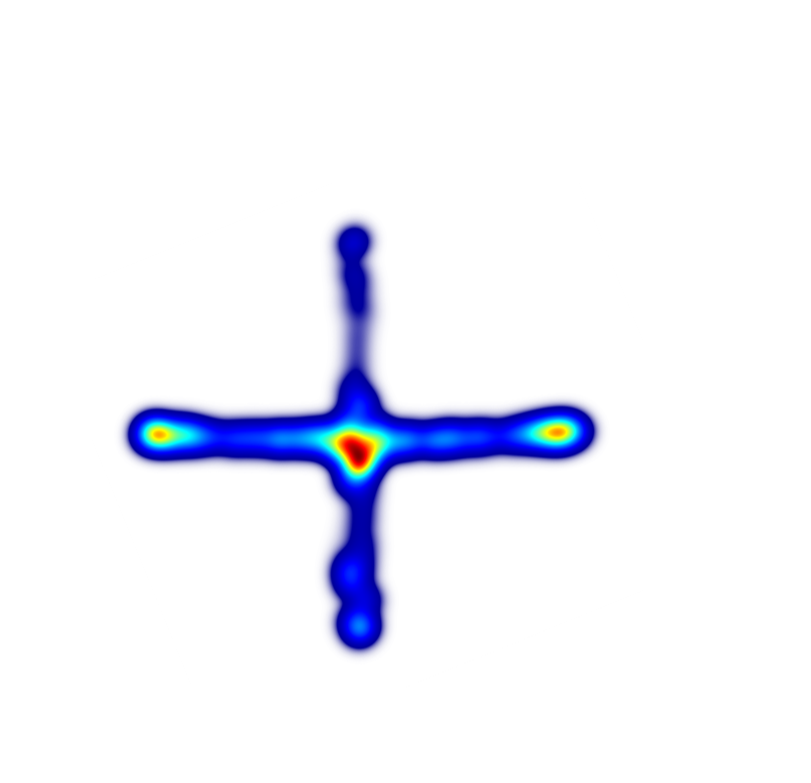

Supplement: Supplementary file 16 — Figure EV Source Data [file 44319_2025_528_MOESM16_ESM.zip › Figure EV4 Source Data/Figure EV4 F left.tif]

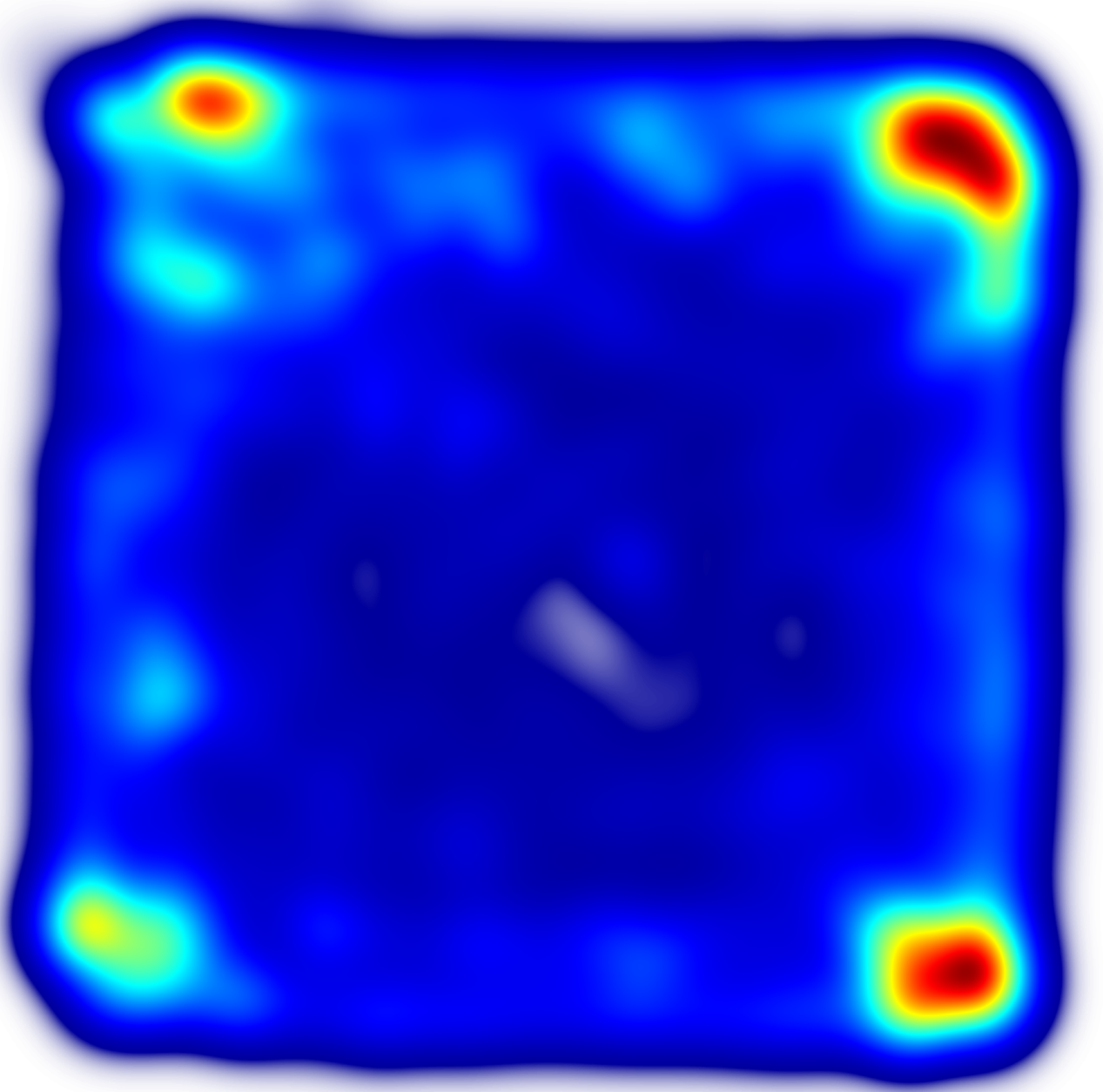

Supplement: Supplementary file 16 — Figure EV Source Data [file 44319_2025_528_MOESM16_ESM.zip › Figure EV4 Source Data/Figure EV4 K left.tif]

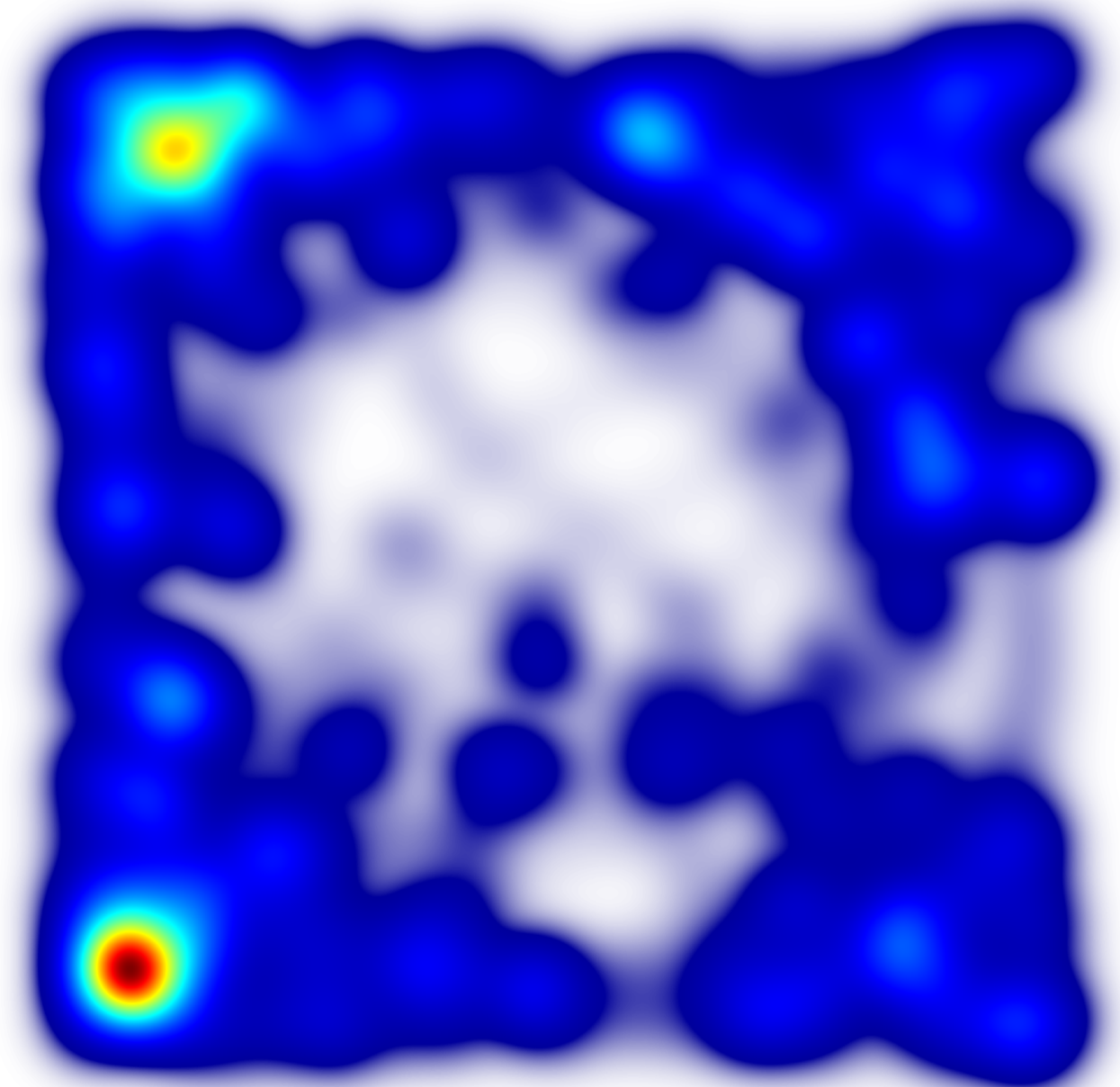

Supplement: Supplementary file 16 — Figure EV Source Data [file 44319_2025_528_MOESM16_ESM.zip › Figure EV4 Source Data/Figure EV4 K right.tif]

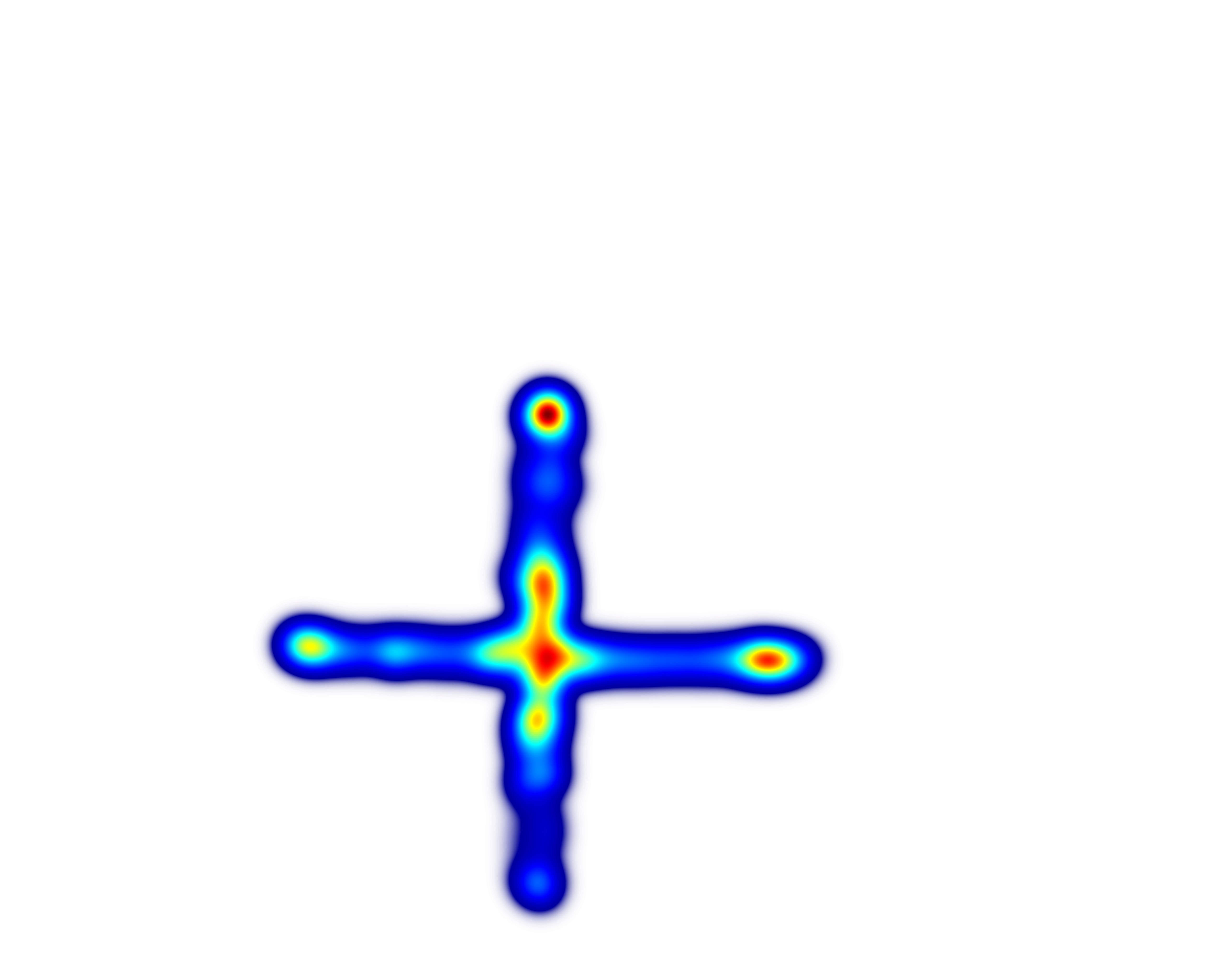

Supplement: Supplementary file 16 — Figure EV Source Data [file 44319_2025_528_MOESM16_ESM.zip › Figure EV4 Source Data/Figure EV4 F right.tif]

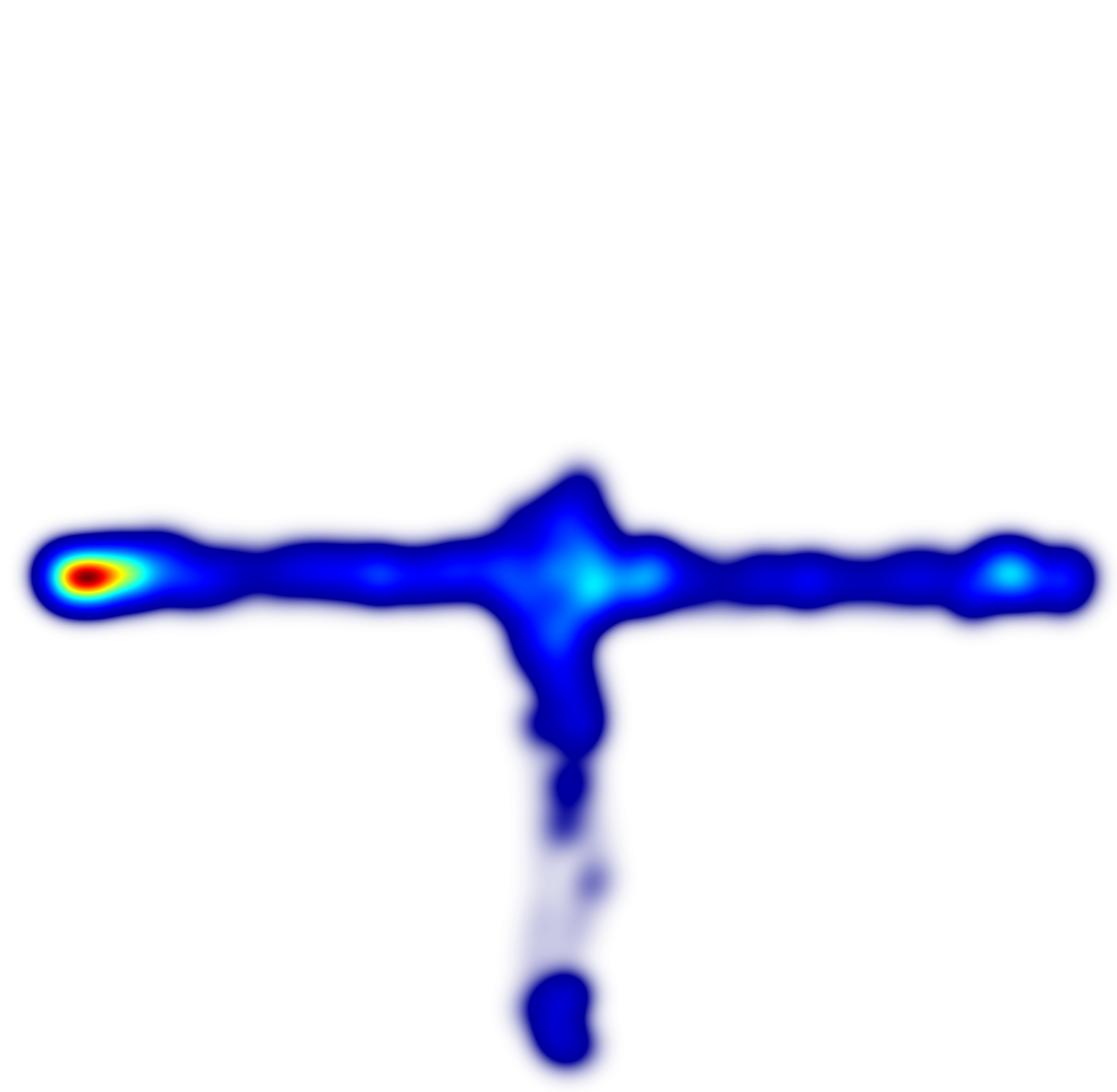

Supplement: Supplementary file 16 — Figure EV Source Data [file 44319_2025_528_MOESM16_ESM.zip › Figure EV4 Source Data/Figure EV4 N right.tif]

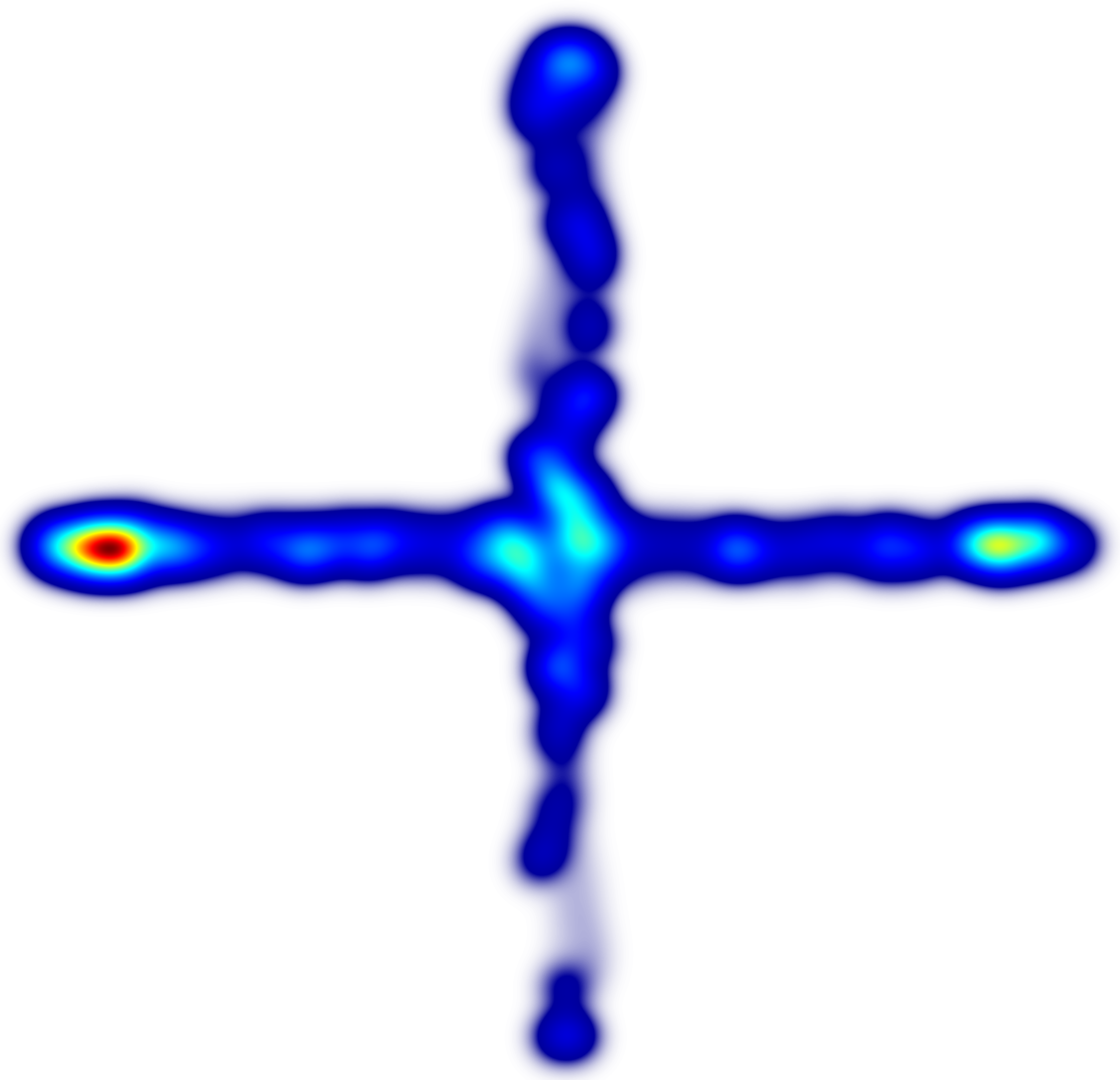

Supplement: Supplementary file 16 — Figure EV Source Data [file 44319_2025_528_MOESM16_ESM.zip › Figure EV4 Source Data/Figure EV4 N left.tif]

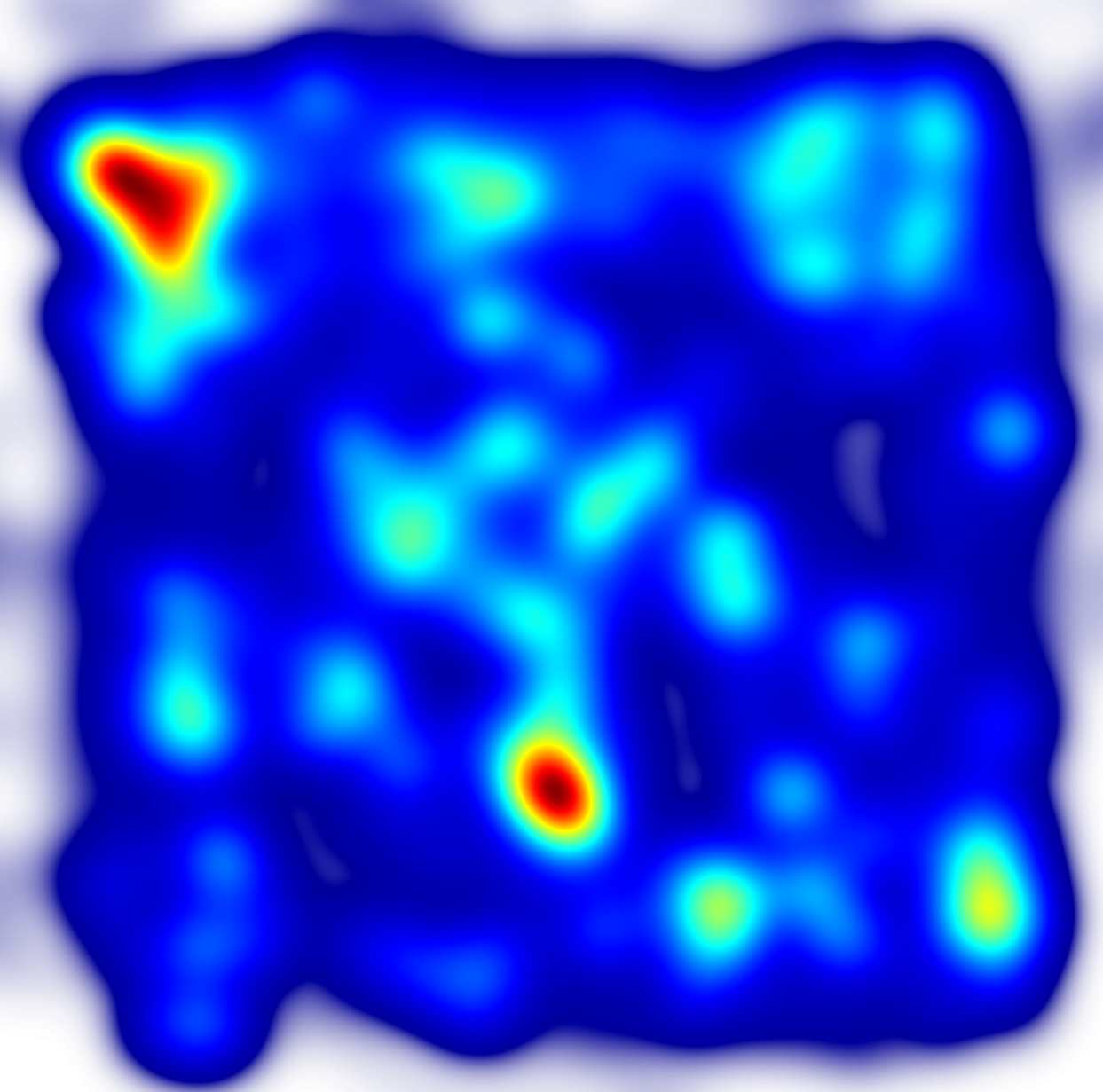

Supplement: Supplementary file 16 — Figure EV Source Data [file 44319_2025_528_MOESM16_ESM.zip › Figure EV4 Source Data/Figure EV4 C right.tif]

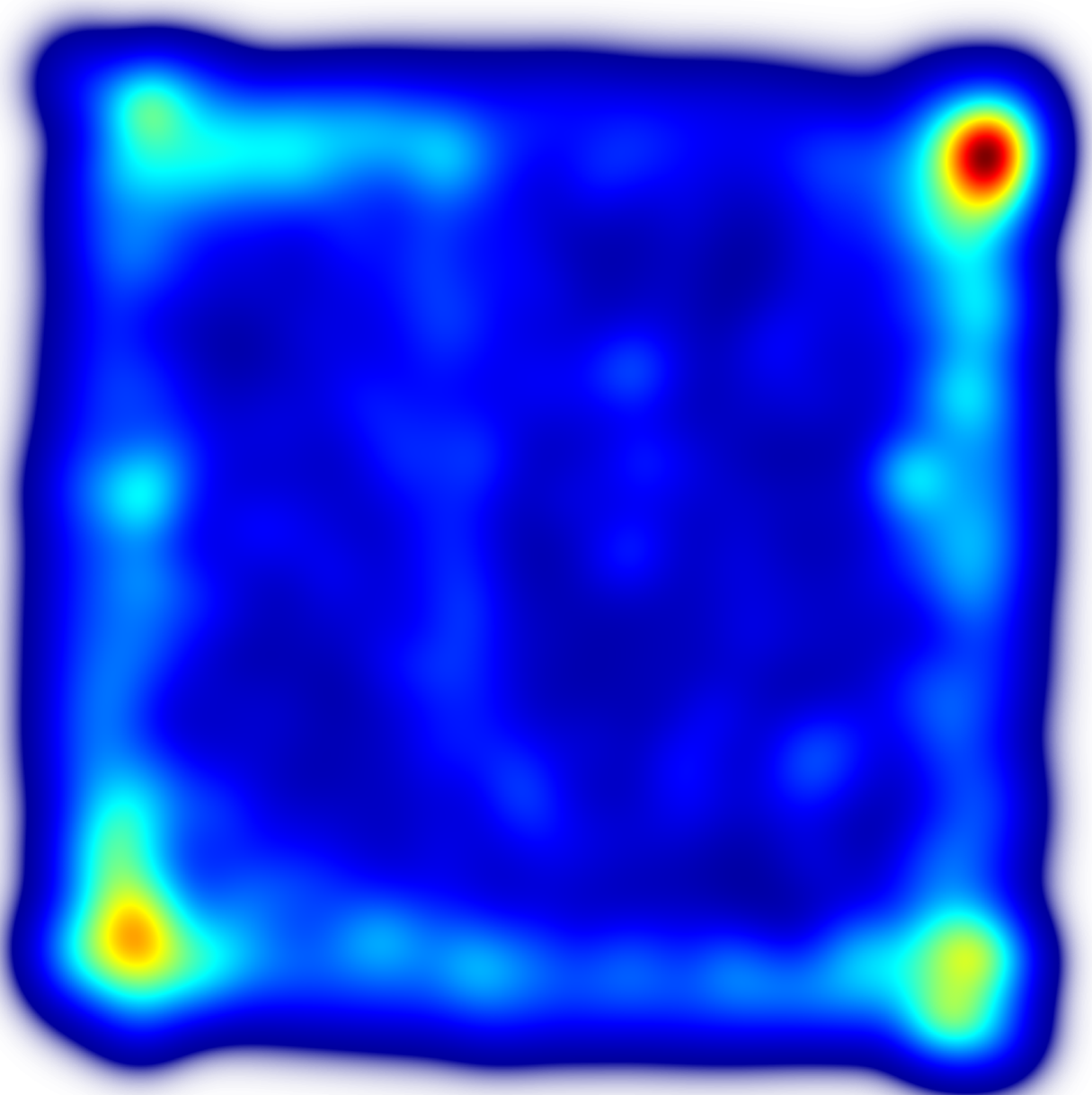

Supplement: Supplementary file 16 — Figure EV Source Data [file 44319_2025_528_MOESM16_ESM.zip › Figure EV4 Source Data/Figure EV4 C left.tif]

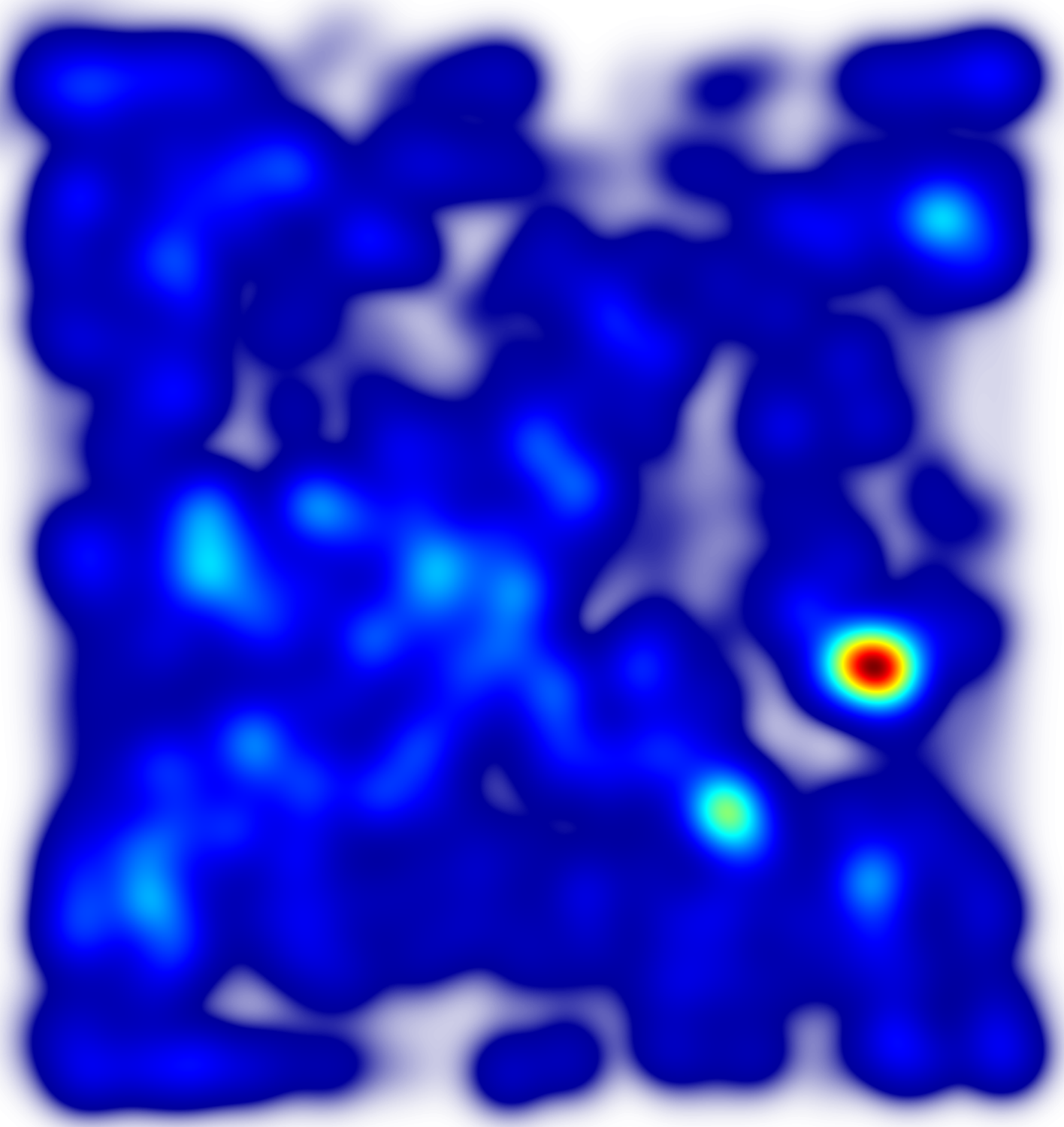

Supplement: Supplementary file 16 — Figure EV Source Data [file 44319_2025_528_MOESM16_ESM.zip › Figure EV7 Data Source/Figure EV7C left.tif]

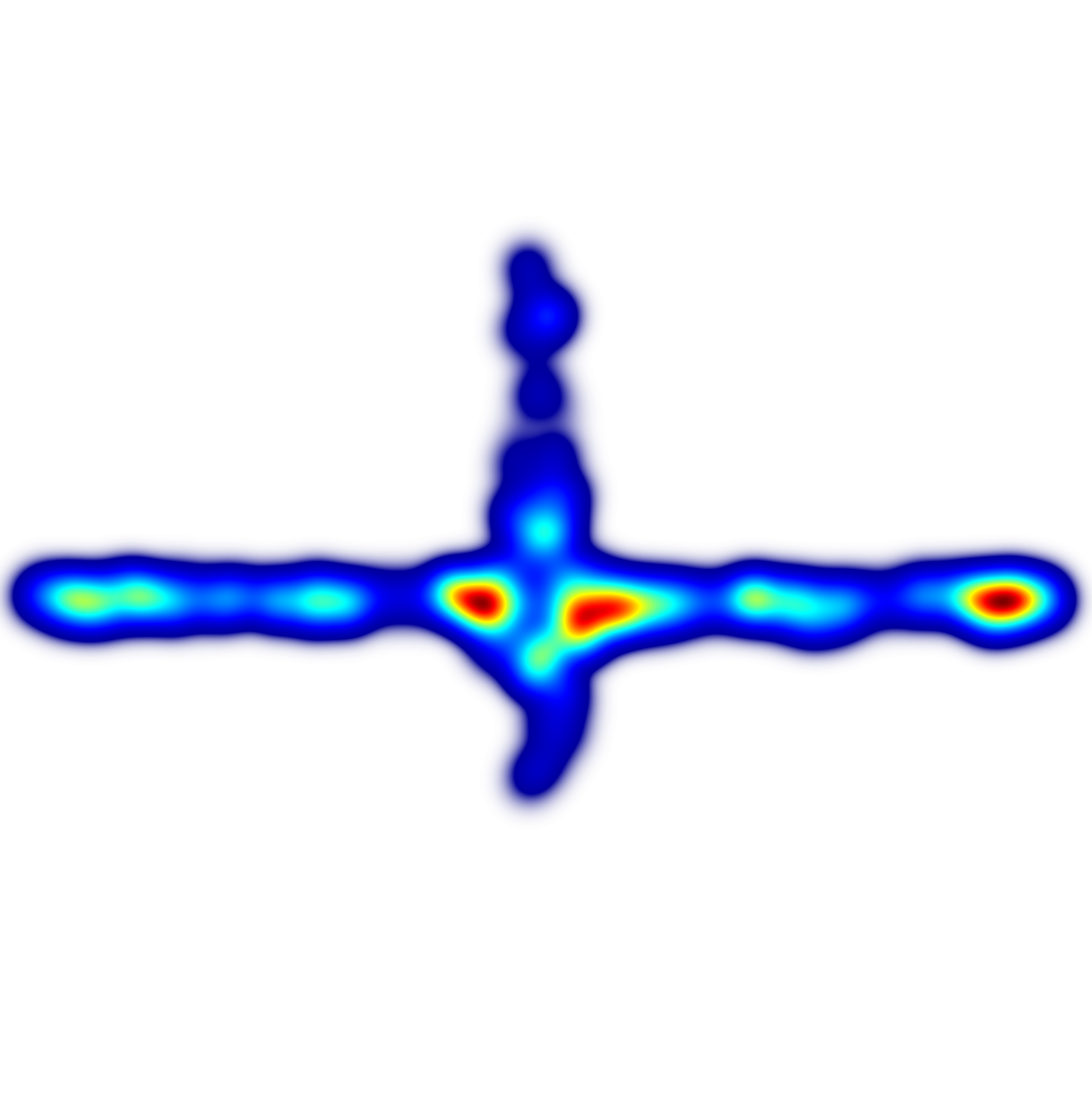

Supplement: Supplementary file 16 — Figure EV Source Data [file 44319_2025_528_MOESM16_ESM.zip › Figure EV7 Data Source/Figure EV7F right.tif]

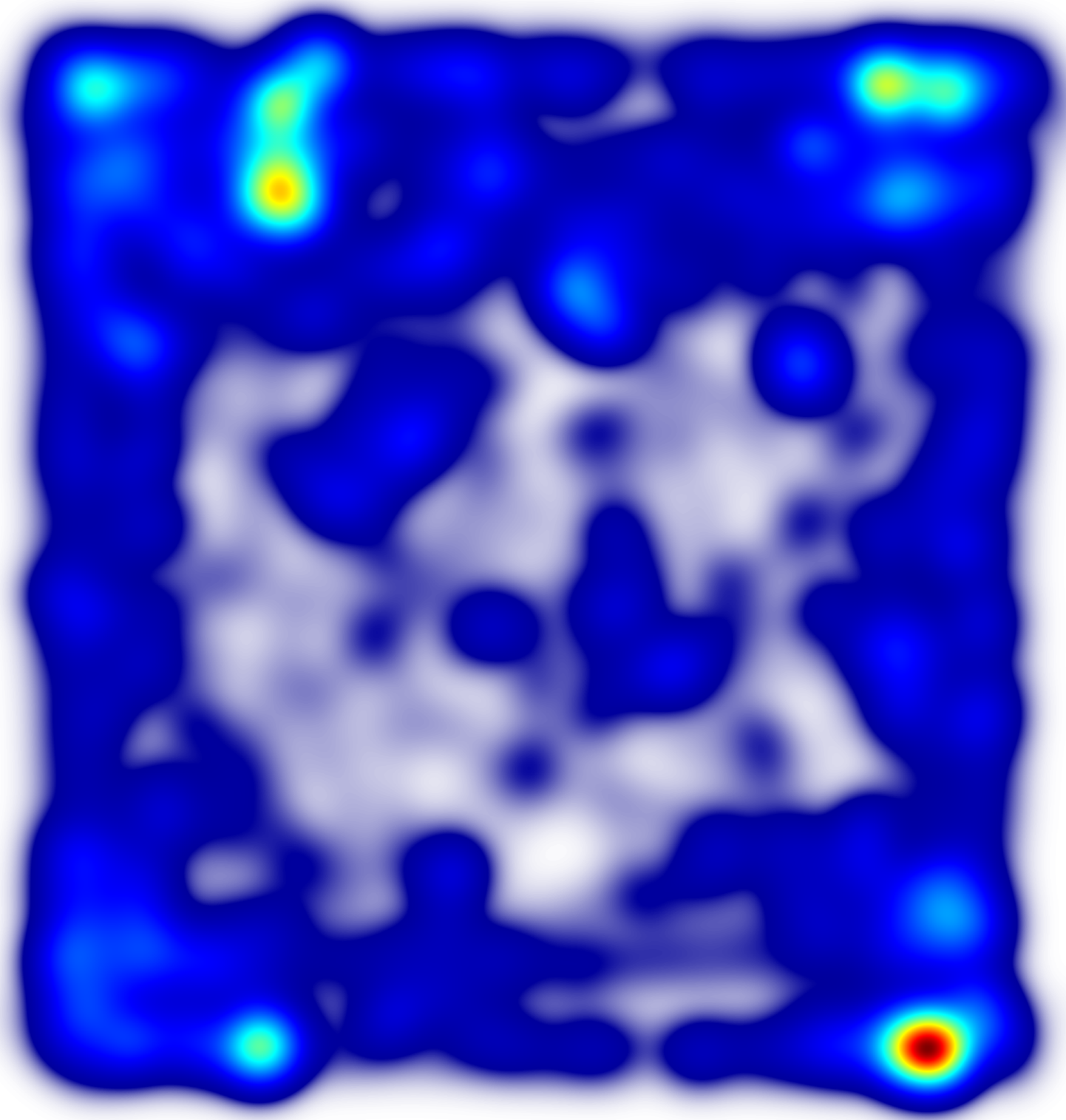

Supplement: Supplementary file 16 — Figure EV Source Data [file 44319_2025_528_MOESM16_ESM.zip › Figure EV7 Data Source/Figure EV7C right.tif]

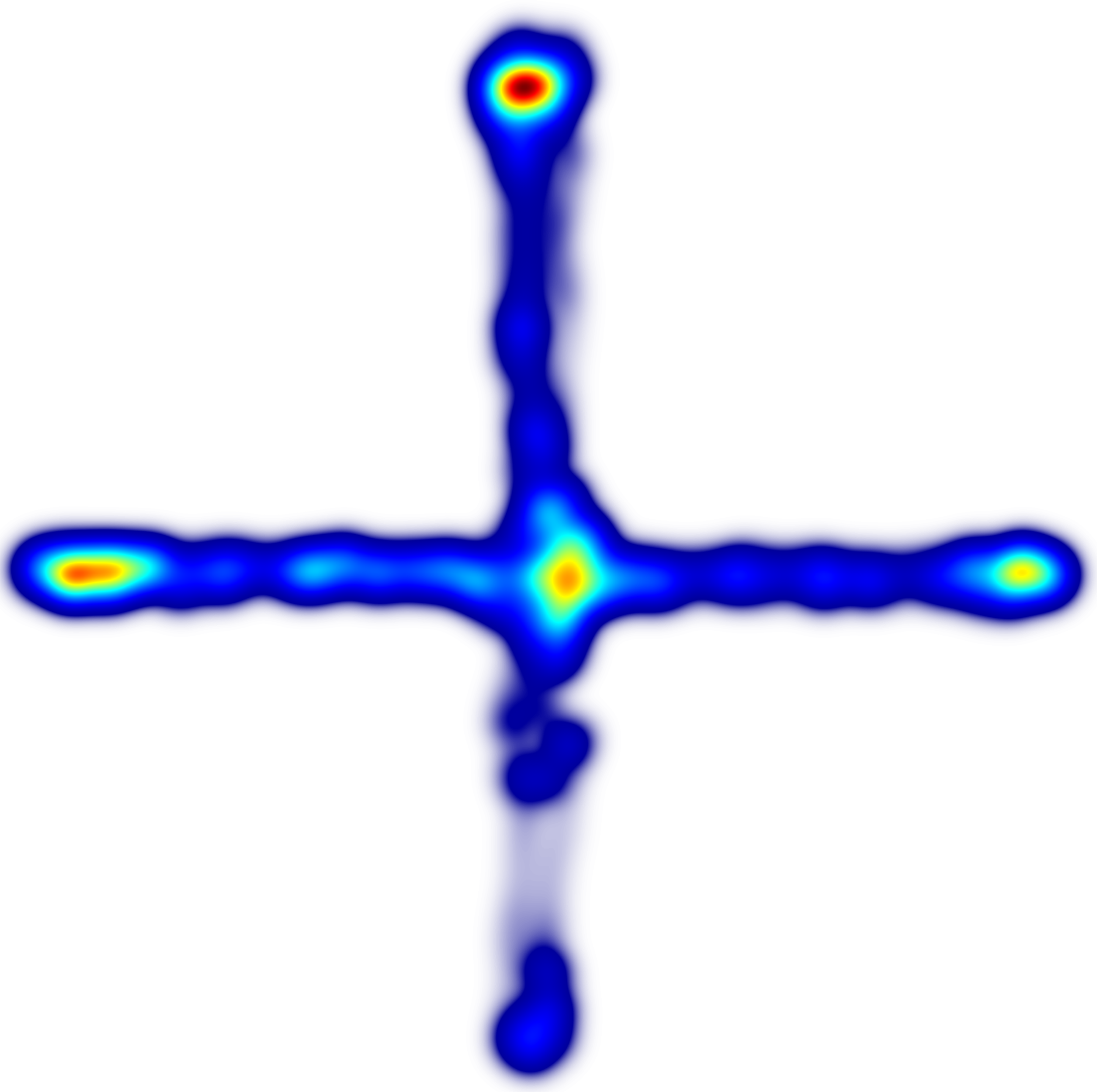

Supplement: Supplementary file 16 — Figure EV Source Data [file 44319_2025_528_MOESM16_ESM.zip › Figure EV7 Data Source/Figure EV7F left.tif]
