# Supplementary material for: A MeA Tac1 neural circuit mediates anxiety-like behaviors in mice
Source: EMBO Rep. 2025 Jul 28;26(17):4340–63. doi: 10.1038/s44319-025-00528-z (PMC12420787; doi:10.1038/s44319-025-00528-z)
Supplement: Supplementary file 17 — Expanded View Figures [file 44319_2025_528_MOESM17_ESM.pdf]

Expanded View Figures

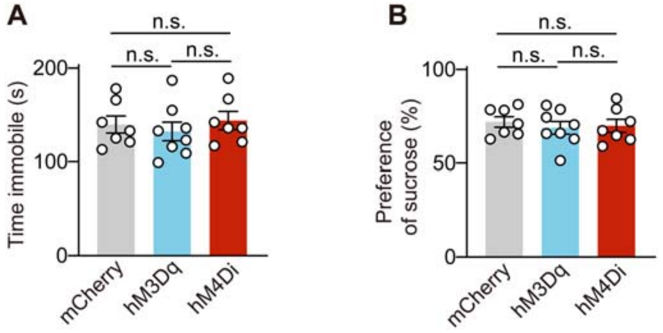

Figure EV1. MeATac1 neurons do not modulate depressive-like behaviors in male mice.

(**A**) The immobile time in the tail suspension test. (**B**) The preference score in the sucrose preference test. mCherry: $N = 7$; M3Dq: $N = 8$; hM4Di: $N = 7$. N animal number. All data are means ± s.e.m. n.s. not significant. See Table EV1 for detailed statistics. Source data are available online for this figure.

 © The Author(s)

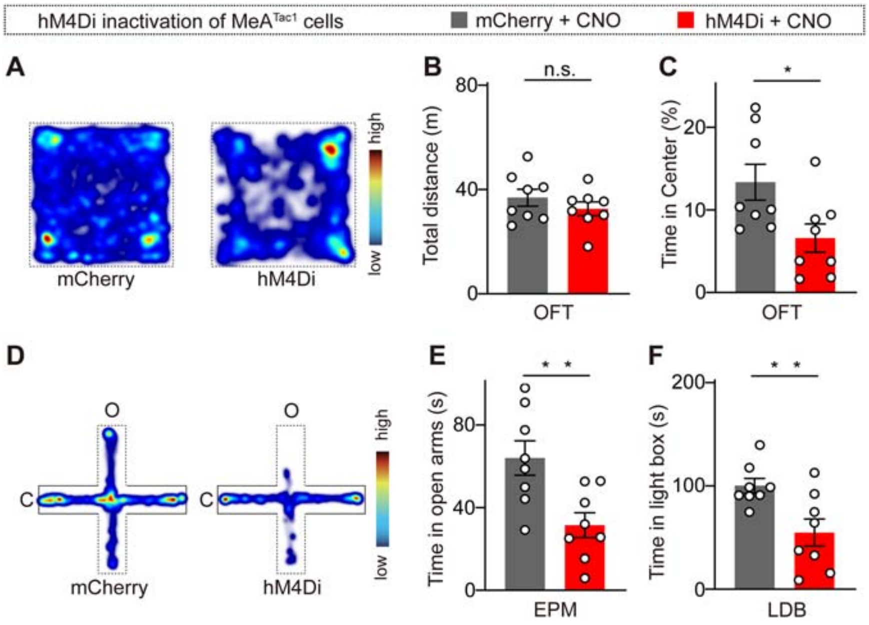

Figure EV2. MeATac1 neurons mediate anxiety-like behaviors in female mice.

(**A**) Heatmaps display time spent in different regions of the open field arena (warmer colors indicate more time). (**B**) Total distance traveled in the open field arena. $P = 0.3245$. mCherry: $N = 8$; hM4Di: $N = 8$. (**C**) Decreased time spent in the center of the open field arena was observed in hM4Di female mice. $P = 0.0275$. mCherry: $N = 8$; hM4Di: $N = 8$. (**D**) Heatmaps display time spent in different regions of the elevated plus maze (warmer colors indicate more time). (**E**) Decreased time spent in the open arms was observed in hM4Di female mice. $P = 0.0068$. mCherry: $N = 8$; hM4Di: $N = 8$. (**F**) Decreased time spent in the light box was observed in hM4Di female mice. $P = 0.0088$. mCherry: $N = 8$; hM4Di: $N = 8$. N animal number. Statistical significance was determined using an unpaired t test. All data are means ± s.e.m. $^*P < 0.05$; $^{**}P < 0.01$; n.s. not significant. See Table EV1 for detailed statistics. Source data are available online for this figure.

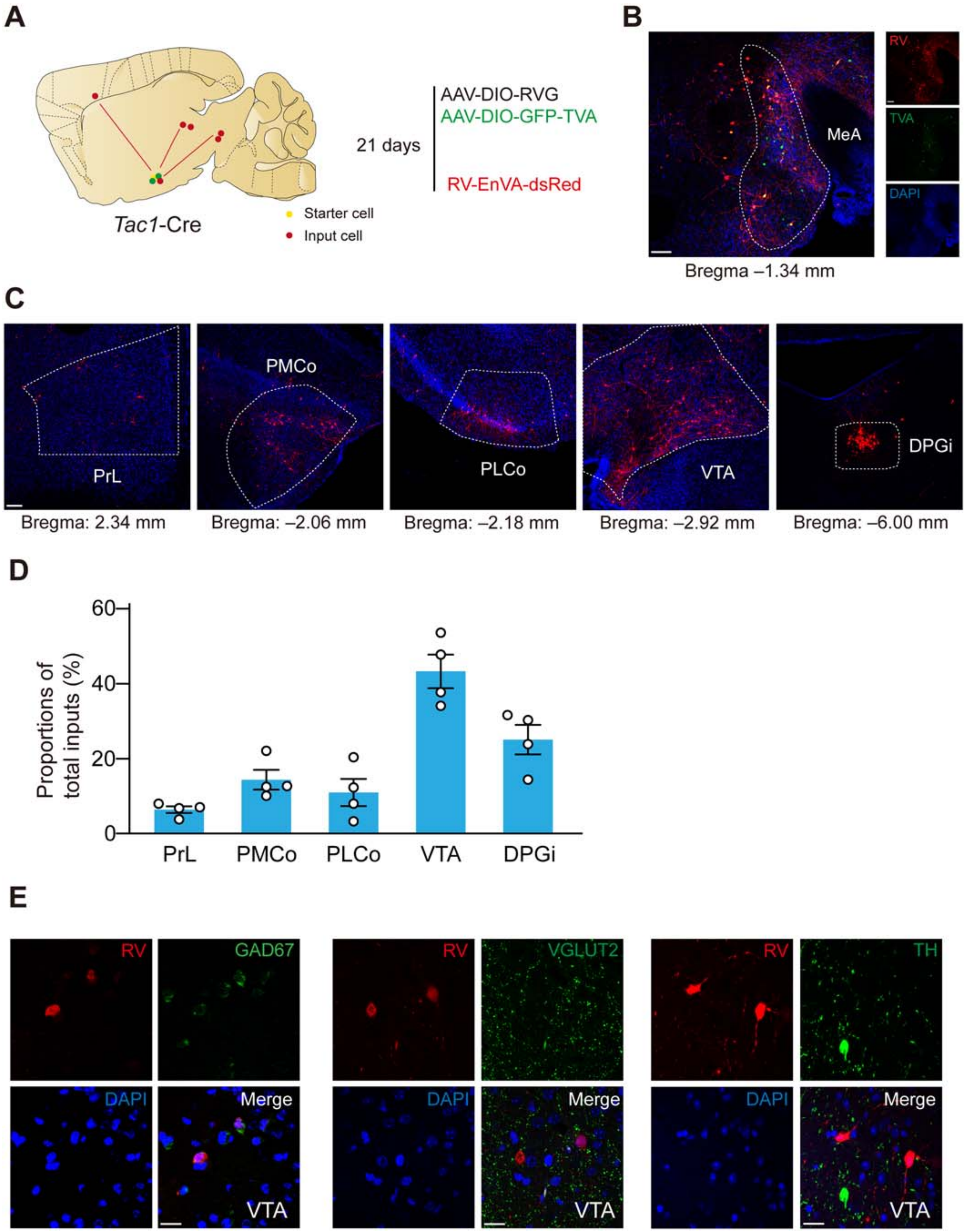
A
Tac1-Cre
Starter cell
Input cell
21 days
AAV-DIO-RVG
AAV-DIO-GFP-TVA
RV-EnVA-dsRed
B
MeA
Bregma –1.34 mm
RV
TVA
DAPI
C
PrL
PMCo
PLCo
VTA
DPGi
Bregma: 2.34 mm
Bregma: –2.06 mm
Bregma: –2.18 mm
Bregma: –2.92 mm
Bregma: –6.00 mm
D
Proportions of total inputs (%)
60
40
20
0
PrL
PMCo
PLCo
VTA
DPGi
E
RV
GAD67
DAPI
Merge
VTA
RV
VGLUT2
DAPI
Merge
VTA
RV
TH
DAPI
Merge
VTA

 © The Author(s)

◀ **Figure EV3. Whole-brain inputs to Tac1 neurons in the MeA.**

(**A**) Schematic of rabies-based cell type-specific monosynaptic tracing procedure. (**B**) Starter neurons with AAV-EF1α-DIO-EGFP-T2A-TVA, AAV-EF1α-DIO-oRVG and RV-ENVA-ΔG-mCherry. Scale bar, 100 μm. (**C**) Representative images of tracing inputs from selected brain regions to MeATac1 neurons. PrL prelimbic cortex, PMCo posteromedial cortical amygdaloid area, PLCo posterlateral cortical amygdaloid area, VTA ventral tegmental area, DPGi dorsal paragigantocellular nucleus. Scale bar, 100 μm. (**D**) Whole brain mapping quantitation of inputs to MeATac1 neurons. $N = 4$. N animal number. (**E**) Representative images of acute slices injected with RV-ENVA-ΔG-mCherry staining with antibodies against GAD67, VGLUT2 and TH. Scale bar, 10 μm. Error bars represent s.e.m. Source data are available online for this figure.

© The Author(s)

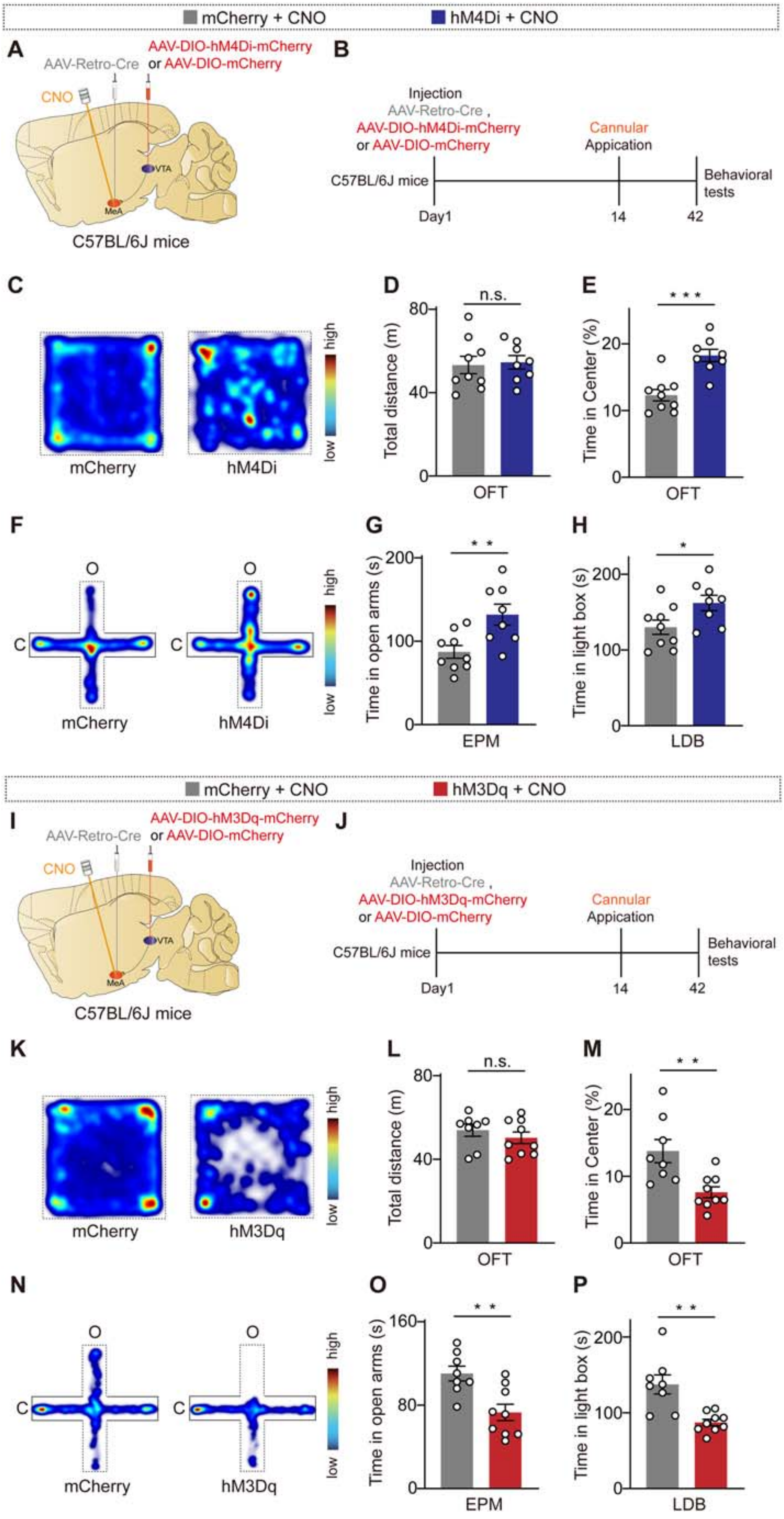
mCherry + CNO
hM4Di + CNO
A
AAV-Retro-Cre
AAV-DIO-hM4Di-mCherry
or AAV-DIO-mCherry
CNO
VTA
MeA
C57BL/6J mice
B
Injection
AAV-Retro-Cre ,
AAV-DIO-hM4Di-mCherry
or AAV-DIO-mCherry
Cannular
Appication
C57BL/6J mice
Behavioral
tests
Day1
14
42
C
mCherry
hM4Di
high
low
D
n.s.
Total distance (m)
OFT
E
Time in Center (%)
OFT
F
O
C
mCherry
hM4Di
G
Time in open arms (s)
EPM
H
Time in light box (s)
LDB
mCherry + CNO
hM3Dq + CNO
I
AAV-Retro-Cre
AAV-DIO-hM3Dq-mCherry
or AAV-DIO-mCherry
CNO
VTA
MeA
C57BL/6J mice
J
Injection
AAV-Retro-Cre ,
AAV-DIO-hM3Dq-mCherry
or AAV-DIO-mCherry
Cannular
Appication
C57BL/6J mice
Behavioral
tests
Day1
14
42
K
mCherry
hM3Dq
L
n.s.
Total distance (m)
OFT
M
Time in Center (%)
OFT
N
O
C
mCherry
hM3Dq
O
Time in open arms (s)
EPM
P
Time in light box (s)
LDB

© The Author(s)

Figure EV4. The VTA→MeATac1 pathway modulates anxiety-like behaviors.

(**A**, **B**) Schematic of the strategies that were used to express AAV-Retro-Cre in the MeA and AAV-DIO-mCherry or AAV-DIO-hM4Di-mCherry in the VTA of C57BL/6 J mice. A cannula was then implanted into the MeA. (**C**) Heatmaps displaying the time spent in different regions of the open field arena (warmer colors indicate more time spent in a region). (**D**) Total distance traveled in the open field arena. $P = 0.8089$. mCherry: $N = 9$; hM3Dq: $N = 8$. (**E**) Time spent in the center of the arena. $P = 0.003$. mCherry: $N = 9$; hM3Dq: $N = 8$. (**F**) Heatmaps displaying the time spent in different regions of the elevated plus maze (warmer colors indicate more time spent in a region). (**G**) Time spent in the open arms. $P = 0.0068$. mCherry: $N = 9$; hM3Dq: $N = 8$. (**H**) Time spent in the light box. $P = 0.0345$. mCherry: $N = 9$; hM3Dq: $N = 8$. (**I**, **J**) Schematic of the strategies that were used to express AAV-Retro-Cre in the MeA and AAV-DIO-mCherry or AAV-DIO-hM3Dq-mCherry in the VTA of C57BL/6 J mice. A cannula was then implanted into the MeA. (**K**) Heatmaps displaying the time spent in different regions of the open field arena (warmer colors indicate more time spent in a region). (**L**) Total distance traveled in the open field arena. $P = 0.3819$. mCherry: $N = 8$; hM4Di: $N = 9$. (**M**) Time spent in the center of the arena. $P = 0.0047$. mCherry: $N = 8$; hM4Di: $N = 9$. (**N**) Heatmaps displaying the time spent in different regions of the elevated plus maze (warmer colors indicate more time spent in a region). (**O**) Time spent in the open arms. $P = 0.0032$. mCherry: $N = 8$; hM4Di: $N = 9$. (**P**) Time spent in the light box. $P = 0.0012$. mCherry: $N = 8$; hM4Di: $N = 9$. N animal number. Statistical significance was determined via an unpaired t test. All of the data are presented as the means ± s.e.m.s. $^{*}P < 0.05$; $^{**}P < 0.01$; $^{***}P < 0.001$; n.s., not significant. See Table EV1 for detailed statistics. Source data are available online for this figure.

© The Author(s)

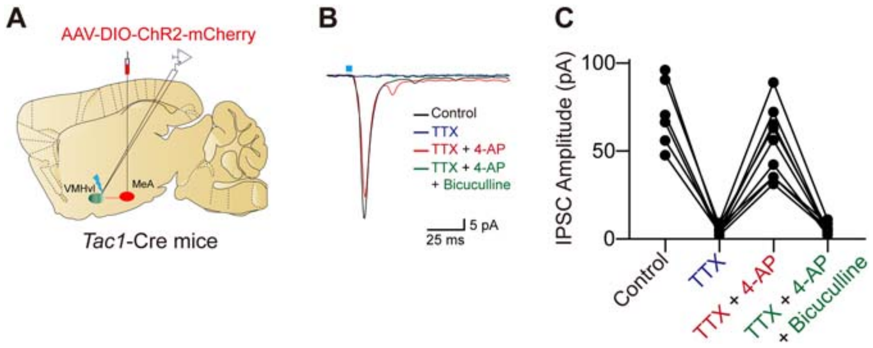

Figure EV5. Identifying the neurotransmission of MeATac1→VMHvl pathway.

(**A**) Schematic of optogenetic electrophysiological recording procedure. (**B**, **C**) Representative traces and quantification of the amplitudes. $n = 8$ cells from 4 animals. MeA medial amygdala. Source data are available online for this figure.

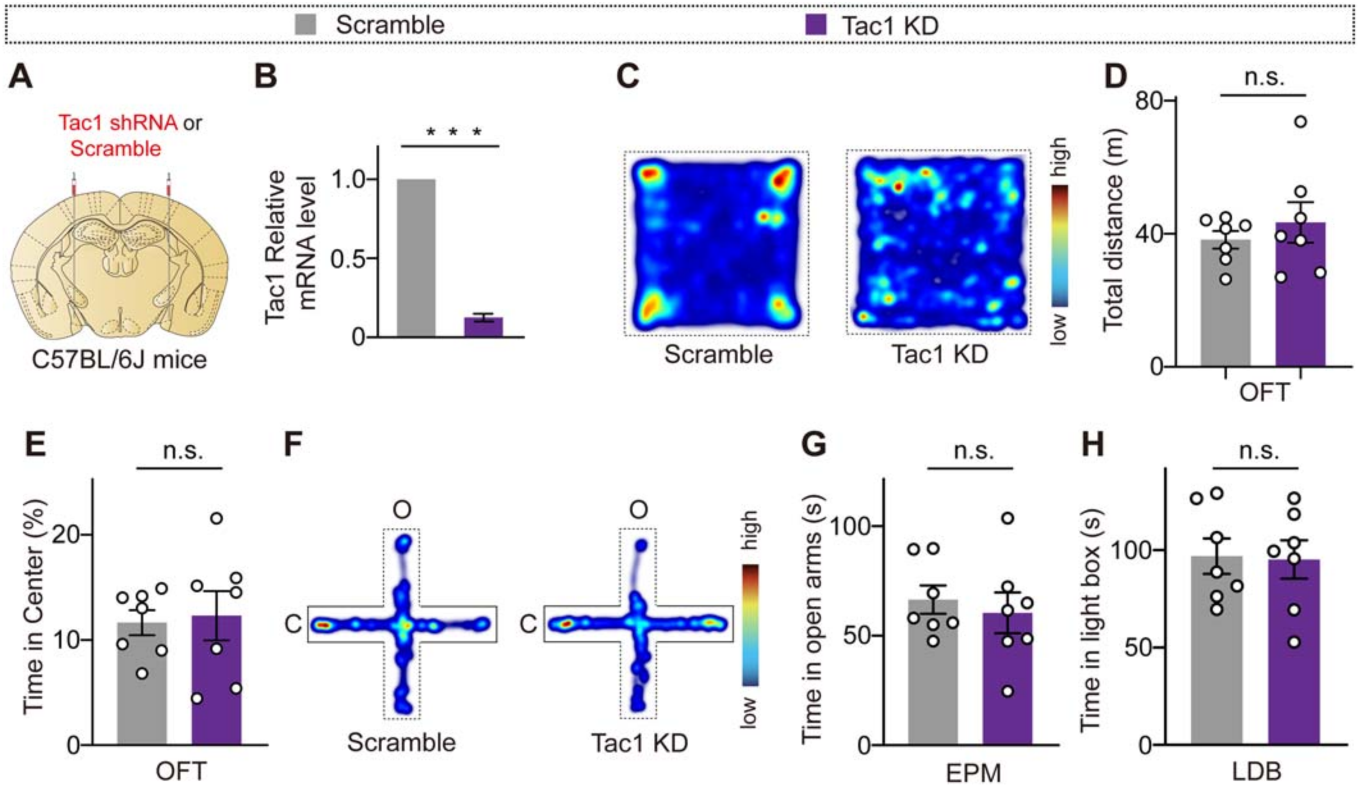

Figure EV6. Substance P does not affect anxiety-like behaviors in mice.

(**A**) Schematic of strategies used to express AAV-Tac1-shRNA and scramble shRNA in C57BL/6J mice. (**B**) Quantitative RT–PCR confirmed the reduction in Tac1 after knockdown. (**C**) Heatmaps display time spent in different regions of the open field arena (warmer colors indicate more time). (**D**) Total distance traveled in the open field arena. $P = 0.4478$. Scramble: $N = 7$; Knockdown: $N = 7$. (**E**) Time spent in the center of the open field arena. $P = 0.8068$. Scramble: $N = 7$; Knockdown: $N = 7$. (**F**) Heatmaps display time spent in different regions of the elevated plus maze (warmer colors indicate more time). (**G**) Time spent in the open arms. $P = 0.6055$. Scramble: $N = 7$; Knockdown: $N = 7$. (**H**) Time spent in the light box. $P = 0.8986$. Scramble: $N = 7$; Knockdown: $N = 7$. Statistical significance was determined using an unpaired *t* test. All data are means ± s.e.m. n.s., not significant. See Table EV1 for detailed statistics. Source data are available online for this figure.

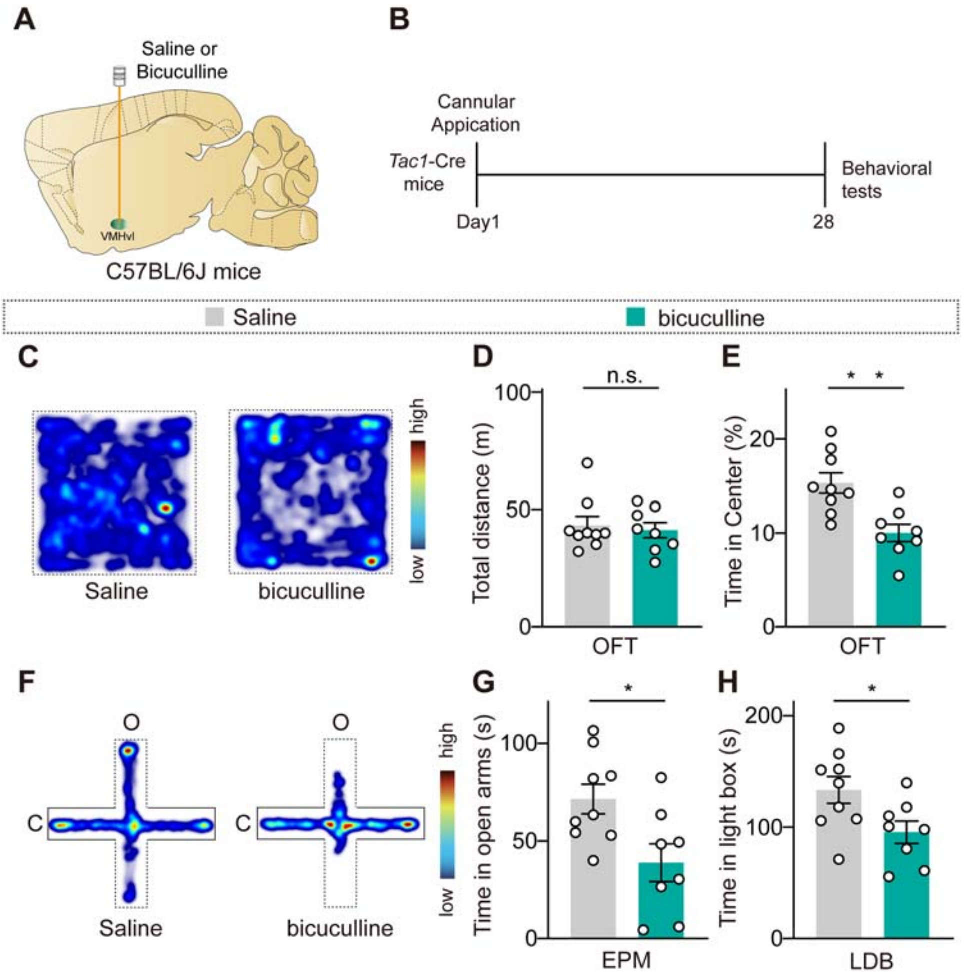

Figure EV7. GABA regulates anxiety-like behaviors in mice.

(**A**) Schematic of experimental strategies. (**B**) Experimental timeline. (**C**) Heatmaps display time spent in different regions of the open field arena (warmer colors indicate more time). (**D**) Total distance traveled in the open field arena. $P = 0.6904$. saline: $N = 9$; bicuculline: $N = 8$. (**E**) Time spent in the center of the arena. $P = 0.0024$. saline: $N = 9$; bicuculline: $N = 8$. (**F**) Heatmaps display time spent in different regions of the elevated plus maze (warmer colors indicate more time). (**G**) Time spent in the open arms. $P = 0.0171$. saline: $N = 9$; bicuculline: $N = 8$. (**H**) Time spent in the light box. $P = 0.0308$. saline: $N = 9$; bicuculline: $N = 8$. N animal number. Statistical significance was determined using an unpaired t test. All data are means ± s.e.m. *$P < 0.05$; **$P < 0.01$; n.s., not significant. See Table EV1 for detailed statistics. Source data are available online for this figure.

 © The Author(s)
